# Engagement and Clinical Improvement Among Older Adult Primary Care Patients Using a Mobile Intervention for Depression and Anxiety: Case Studies

**DOI:** 10.2196/16341

**Published:** 2020-07-08

**Authors:** L Casey Orr, Andrea K Graham, David C Mohr, Carolyn J Greene

**Affiliations:** 1 Center for Health Services Research Psychiatric Research Institute University of Arkansas for Medical Sciences Little Rock, AR United States; 2 Center for Behavioral Intervention Technologies Northwestern University Chicago, IL United States

**Keywords:** mobile health, older adults, depression, anxiety, primary care, smartphone, mobile phone, text messaging

## Abstract

**Background:**

Technology-based mental health interventions are an increasingly attractive option for expanding access to mental health services within the primary care system. Older adults are among the groups that could potentially benefit from the growing ubiquity of technology-based mental health interventions; however, older adults are perceived to be averse to using technology and have reported barriers to use.

**Objective:**

The aim of this paper is to present a case study of 3 participants from a clinical trial evaluating IntelliCare, an evidence-based mobile intervention for depression and anxiety, among adults recruited from primary care clinics. Our report of these 3 participants, who were aged 60 years or older, focuses on their engagement with the IntelliCare service (ie, app use, coach communication) and clinical changes in depression or anxiety symptoms over the intervention period.

**Methods:**

The 3 case study participants were offered IntelliCare with coaching for 8 weeks. The intervention consisted of 5 treatment intervention apps that support a variety of psychological skills, a *Hub* app that contained psychoeducational content and administered weekly assessments, and coaching for encouragement, accountability, and technical assistance as needed. The 3 case study participants were selected to reflect the overall demographics of participants within the trial and because their interactions with IntelliCare provided a good illustration of varied experiences regarding engagement with the intervention.

**Results:**

The 3 participants’ unique experiences with the intervention are described. Despite potential barriers and experiencing some technical glitches, the participants showed proficient ability to use the apps, high levels of participation through frequent app use and coach interaction, and decreased depression and anxiety scores. At the end of the 8-week intervention, each of these 3 participants expressed great enthusiasm for the benefit of this program through feedback to their coach, and they each identified a number of ways they had seen improvements in themselves.

**Conclusions:**

These 3 cases provide examples of older individuals who engaged with and benefitted from the IntelliCare service. Although the results from these 3 cases may not generalize to others, they provide an important, informed perspective of the experiences that can contribute to our understanding of how older adults use and overcome barriers to mental health technologies. The findings also contribute toward the ultimate goal of ensuring that the IntelliCare intervention is appropriate for individuals of all ages.

## Introduction

### Background

There is a pressing need for accessible and effective mental health treatment within the primary care system [1]. Typically, primary care is the first and often only point of contact for those with mental health conditions, particularly anxiety and depression, as opposed to specialty mental health professionals and clinics [2]. However, access to adequate mental health treatment remains limited. Scarce provider resources and varied geographic, economic, and health factors hinder patients’ ability to access treatment [3]. Those who do receive behavioral health treatment through primary care face barriers to adequate services [4]. As a result, technology-based mental health interventions are an increasingly attractive option.

Older adults are among the groups that could potentially benefit from the growing ubiquity of technology-based mental health interventions. Unfortunately, popular culture and conventional beliefs suggest that technology-based interventions are targeted toward younger people, with the perception that older people are averse to using technology [5,6]. Indeed, it has been shown that older adults are less likely to use internet-enabled technologies and smartphones compared with the national average. [7]. Furthermore, older adults have expressed apprehension regarding technology use [8]. Although telehealth and web-based interventions have demonstrated efficacy for addressing depression among older adults [9,10], important barriers remain to engaging older adults in technology-based interventions. Older adults report a variety of reasons for which they avoid using technology for mental health, including a desire for human contact [11]. Another study found that technology utilization among older adults is hindered by cost, complexity, ergonomic impediments, and a lack of interest [12]. Furthermore, design challenges, trust of the technology, familiarity, willingness to ask for help, and privacy have also been identified as barriers to the acceptance and use of health information technology by older adults [13].

### Objectives

We conducted a clinical trial evaluating IntelliCare, an evidence-based mobile intervention for depression and anxiety, among adults recruited from primary care clinics at the University of Arkansas for Medical Sciences (UAMS) [14,15]. IntelliCare has been publicly available since 2014 and has shown favorable results in 2 previous trials, with past users demonstrating high engagement (>90% continued use through 8 weeks of treatment in coached deployments) and substantial improvements in depression symptoms from pre- to postintervention [16,17].

During the enrollment process for the clinical trial, we observed that 13.7% (n=20/146) of those enrolled in the study were aged 60 years or older. Therefore, we sought to understand how these older participants would fare with the IntelliCare intervention. Thus, as an initial step in this research, we conducted a case study of 3 enrolled participants aged 60 years and older, focusing specifically on their engagement with the IntelliCare service (ie, app usage, coach communication) and clinical changes in depression and anxiety symptoms over the intervention period. Our objective was to illuminate these 3 individuals’ aptitude for using the IntelliCare intervention to improve their mental health, toward the ultimate goal of ensuring this intervention is appropriate for individuals of all ages.

## Methods

### Participants

This is a case study report of 3 participants of the 146 adults enrolled in a randomized controlled trial investigating the efficacy of the IntelliCare intervention among primary care patients with depression and/or anxiety [15]. The UAMS Institutional Review Board approved this study, and all participants provided informed consent.

To be included in the IntelliCare trial, participants presented to UAMS primary care within the past year; had elevated symptoms of depression or anxiety at screening, defined as a score 10 on the Patient Health Questionnaire-8 (PHQ-8), which excluded the item assessing suicidality [18], or a score 8 on the 7-item Generalized Anxiety Disorder scale (GAD-7) [19]; and had a compatible smartphone with an SMS text message and data plan. Participants were excluded if they were experiencing high suicidal risk (ie, had ideation, plan, and intent) on the baseline assessment, were currently receiving psychotherapy, had recent changes in psychotropic medication, had a psychiatric condition not suitable for participation, or had a visual, motor, or hearing impairment that prevented participation in study procedures. Enrolled participants were randomized to receive either the IntelliCare intervention immediately or after an 8-week waitlist period. As part of the research assessments, participants completed the PHQ-9 [20] and GAD-7 at baseline and every 4 weeks for 16 weeks.

For the 3 case studies, we observed 2 white participants and 1 African American participant, two of whom were identified as female and one as male. This subset reflects the overall demographics of participants within this trial, who were identified as 65.1% (n=95/119) white and 32.2% (n=47/146) African American and the majority (n=119/146, 81.5%) of whom were identified as female. All 3 case study participants were aged 60 years or older. We chose these 3 participants because their interactions with IntelliCare provided a good illustration of varied experiences regarding engagement with the intervention and overcoming previously identified barriers. More specifically, the participants who were selected highlighted specific instances of the ability to overcome barriers to technology use for mental and physical health among older adults identified in previous studies [11-13], which were observed through interactions with their coach during which these barriers were discussed. Participants not chosen for this case study spoke less of their experiences using the intervention and therefore provided less insight into their interactions with the technology.

### The IntelliCare Intervention

**Figure 1:**
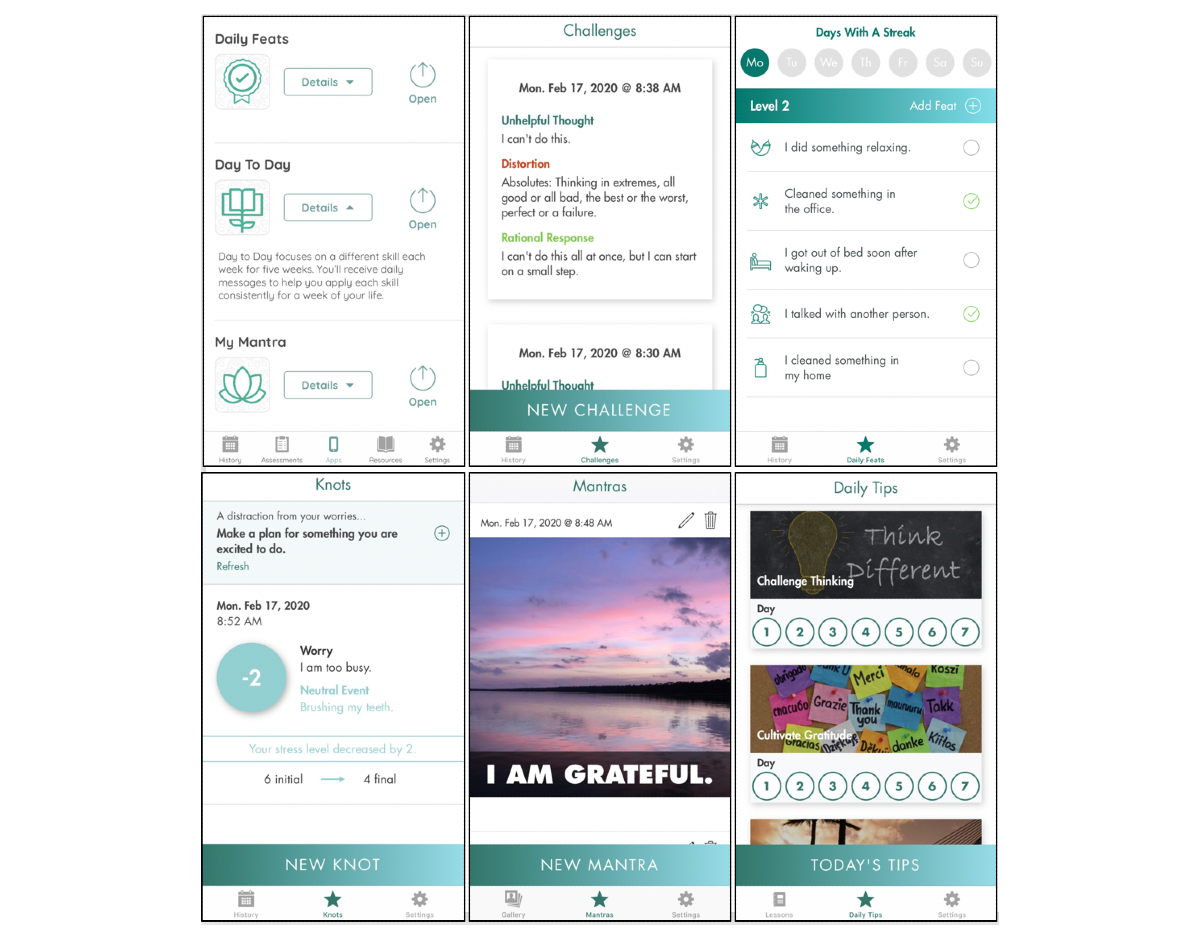
IntelliCare apps. Top row, left to right: *Hub*, *Thought Challenger*, *Daily Feats*. Bottom row, left to right: *Worry Knot*, *My Mantra*, *Day to Day*.

All individuals enrolled in the IntelliCare study were offered the IntelliCare intervention, comprising a suite of mobile apps and live coaching, for 8 weeks. The version of IntelliCare used in this trial included a *Hub* app and 5 intervention apps, each of which were designed to be used in brief, frequent interactions to support a variety of psychological skills. Screenshots of the apps are presented in Figure 1.

The *Hub* app facilitated participants’ engagement by supporting direct download and access to clinical apps, providing a library of information and psychoeducational materials, and administering weekly PHQ-8 surveys. The intervention apps provide basic information about anxiety and depression and employ evidence-based skills for managing symptoms. Each app focuses on a specific skill. The *Thought Challenger* app focuses on cognitive restructuring and helps users to identify unhelpful thoughts, recognize associated cognitive distortions, and substitute unhelpful thoughts with more realistic thoughts. *Daily Feats* focuses on goal setting by helping users create a list of daily goals (ie, *feats*) that they can check off throughout the day; as users complete feats each day, they earn *streaks* from which they can *level up* to get a new list of more advanced goals to accomplish. *Worry Knot* focuses on anxiety exposure. It helps users learn to experience thoughts about the past or future without increasing stress by associating the stressful thought with a neutral thought. *My Mantra* uses positive self-statements and personal pictures from the users’ phone to remind them of their values and the gratifying parts of their lives. Finally, *Day to Day* uses weekly positive psychology lessons tied to daily tips (ie, suggestions) to help challenge thinking, cultivate gratitude, activate pleasure, increase connectedness, and solve problems. Participants were encouraged to use the apps regularly, for a few minutes daily, to gain proficiency in learning the skills and practicing relevant strategies in their daily lives.

#### Coaching

All individuals were assigned a coach. The coaching protocol was defined by the IntelliCare Coaching Manual [21], which is based on the supportive accountability and efficiency models of coaching [22,23]. Coaches were bachelors-level individuals trained to deliver the intervention according to the IntelliCare Coaching Manual and supervised weekly by a licensed clinical psychologist. The coach provided encouragement and accountability for participants to use the apps, helped to problem-solve obstacles, recommended apps that would be most useful, and provided technical assistance as needed. The coach did not deliver psychotherapy but rather served as a guide to help the participant understand the apps and connect the skills to their goals.

#### Intervention Delivery

Upon entering the active intervention phase, participants received a welcome packet and completed a 30- to 45-min introductory telephone call with their coach. During that call, the coach explained the format of the program, established an alliance, identified the participant’s unique goals for treatment, and ensured that the *Hub* app was properly installed on the participant’s phone. The coach also recommended one intervention app to begin using after assessing the participant’s preferences. The participant was encouraged to use the first app for 1 week. Each subsequent week, the coach recommended a new app for the participant to try. Following the initial introductory call, communication was conducted via SMS text messaging. Coaches messaged participants to provide positive reinforcement, encourage exploration of apps, make app recommendations, or problem-solve as issues arise. Participants were free to message the coach at any time and were told that responses would come within one business day (although the response time was often much shorter).

Over the course of the 8 weeks, coaches texted participants weekly, typically initiating 2 contacts per week and responding to participants’ SMS text messages as they came in. Usually, the first SMS text message from the coach at the beginning of each week was used to introduce a new app and provide a brief explanation for how it could be helpful in their daily lives. Afterward, another SMS text message midweek was sent to check in on their interaction with that app. The coach would inquire about any technical issues or barriers to use and ensure that the participant understood how to use the app as intended. At this time, the coach might help the participant brainstorm different areas of their lives to which they could apply the skills, based on the participant’s goals that were discussed during the initial phone call. Sometimes, a technical issue could cause the participant to feel frustrated and discouraged. The coach worked closely with the participant and the technical team to resolve technical issues, whether it was due to user error or a *bug* in the system. If the participant did not like the app, found it uninteresting, or thought it was a poor fit with their goals, the coach would suggest a different app to try. Participants could use as many apps as they saw fit with their needs and could discontinue using any app that did not fit with their goals. After 4 weeks, an optional midpoint telephone call with the coach was offered to check in on the participant’s progress toward goals and identify any barriers to app use.

#### Clinical Management Dashboard

Throughout the 8 weeks, the coach monitored a dashboard that displayed the participant’s app usage, a record of the SMS text messages exchanged, and weekly depression scores. The dashboard also provided additional participant management tools. The coach could set scheduling reminders and utilize a notes section to record each participant’s initial goals and comments during the introductory and midpoint phone calls. The coach could also create a *ticket* to directly alert the technical team of any new issues involving the apps not working properly.

In terms of app usage, the coach could only see how frequently the participant engaged with the apps but could not see the data that were entered into the apps by the participant, except for the PHQ-8 responses. The coach utilized the app usage frequency data to determine if the participant needed encouragement to engage more often with their apps. The coach would also ask participants about their change in mood if the coach observed an increased depression score. This often led to a productive discussion about new goals to work on, life changes, or deeper issues that needed to be addressed by other means—in which case, the coach would talk to the participant about making an appointment with their primary care physician.

## Results

### Case Study 1

Participant A was a 69-year-old white man who was single, retired, and lived in an assisted living home. He reported that his highest level of education was a master’s degree. His presenting issues included concerns about not being active or social enough, having no friends, low motivation, low energy, and trouble falling asleep. His goals for treatment were to be more involved in “something” (eg, volunteering) and attend activities involving others, improve his self-esteem and impressions of his current life stage and situation, and build confidence to go back to church services.

Over the course of the intervention, Mr A consistently communicated with the coach. He almost always responded to the coach’s SMS text messages and frequently initiated SMS text messages to give the coach updates on his activities. He was also highly consistent in using the apps: he used them once or more per day on 52 out of 56 days. Mr A’s frequent and consistent level of app use is particularly notable given that, at the start of the program, he experienced some glitches with the technology that had to be resolved by the technical team. Specifically, he initially experienced technical issues with *Daily Feats*, the first app recommended to him. As this app focused on goal setting and achieving daily behavioral goals, it aligned with what he was hoping to accomplish in treatment. In SMS text message exchanges with his coach, he asked questions about how the app worked. The coach carefully explained the structure of the app, and Mr A showed frequent use throughout the first week. However, in week 2, he began to experience trouble with the tracking system in the app, which he proactively reported to the coach. This demonstrates a willingness to ask for help, a previously identified barrier to health technology use by older adults [13]. He described the issue, and the coach expressed regret for inconvenience and created a ticket describing the incident, which was sent to the technical team. The coach monitored updates from the technical team and reassured Mr A that they were working on the issue. Waiting for the glitch to resolve, the coach switched focus to Mr A’s next app, *Day to Day*, asking about his impression of it and congratulating him for his continued use of both apps. He expressed interest in this new app and noted how the two complement each other: “one is conceptual and the other puts thoughts into action.”

Over the course of the next few weeks, they moved to focus on his next few apps and scheduled a midpoint call. On the call, Mr A explained his continued trouble with the *Daily Feats* app while also discussing the other apps and his progress toward his goals. At the end of week 5, after the technical team had incorporated app updates, the coach asked him about *Daily Feats*. To demonstrate that the problems had been resolved, he shared a screenshot that showed his tracked progress of completed goals. A few days later, he sent an SMS text message saying that he “finally leveled up,” which indicated that he had successfully completed 2 goals each day for at least five days in a row. Taken together, Mr A demonstrated aptitude for engaging with the intervention despite technical issues. Although Mr A initially expressed that these glitches were “disconcerting,” he overcame these obstacles and continued to use the apps on a near daily basis despite the barrier of usability challenges, including confusion about how to use some of the apps [12,13]. In fact, he indicated that *Daily Feats* was his favorite app, which was also reflected in his usage data as his most frequently used app. This shows his ability to overcome the barriers of lack of interest and familiarity with the technology [12,13].

The translation of skills to his daily life was evident through interactions with his coach. During his midpoint phone call at the end of week 4, he reported positive impressions of the intervention and progress toward his treatment goals. He said that he thought the apps were “great” and that the coaching aspect was a key part of the program. He reported that he was trying to be positive about social situations and liked that the apps helped him maintain positive thoughts. He demonstrated progress toward his treatment goals in that he was making plans to attend a community engagement workshop and was saying “yes” to opportunities to be in social situations. From this call, the coach observed a notable change in his tone of voice, which suggested that he had gained a heightened level of confidence and enthusiasm. In week 6, when reflecting on his progress with treatment in an SMS text message to the coach, he shared that it “feels like I’m on the road to wellville.”

At screening, Mr A had elevated depression and anxiety, with PHQ-8 and GAD-7 scores of 16 and 11, respectively. These scores were in the moderately severe depression and moderate anxiety ranges, respectively. When he began the intervention, his PHQ-9 and GAD-7 scores were 13 and 9, respectively. After completing the intervention, his PHQ-9 depression and GAD-7 anxiety scores were both 6 (ie, mild range). These scores are consistent with Mr A’s engagement with the intervention and his reported progress. Although he indicated that he still had challenges with his goal of becoming more social, his communications with the coach revealed that his overall outlook had become more positive, and he said he was making plans to get more involved.

### Case Study 2

Participant B was a 60-year-old African American woman with an associate degree (ie, 2-year college degree), who was married and employed. In the initial coaching call, she reported feeling that she was not doing enough at work or getting things done on time, causing her to feel stressed about falling behind and trying to catch up. She also expressed anxiety about driving in traffic and feelings of sadness that her husband does not spend as much time with her as she would like. Her goals for treatment were to keep thoughts positive, get along better with others, and control negative attitudes.

Over the course of the intervention, Mrs B frequently initiated conversations with the coach. She reliably responded to texts from the coach, sending her replies either the same day or within a few days. She was a moderately high user of the apps; the longest gap without app use was 3 days. Mrs B connected well with the *Thought Challenger* app, saying that it helped her focus on more positive thoughts. A few days after she started using the app, the coach asked if she had been able to identify any patterns in distortions surrounding her negative thoughts. This question prompted a productive SMS text message exchange in which the coach helped Mrs B more clearly understand the concept of cognitive distortions. Mrs B was able to identify her unhelpful thoughts and recognize the cognitive distortions that she commonly experienced. She specifically mentioned that she had a tendency to magnify her negative thoughts, making them a bigger issue than they really are. She felt confident that this knowledge would contribute to her progress in changing her thoughts to become more positive. This scenario provides an example of how the intervention with coaching met the desire for human contact, facilitated rectifying a misunderstanding about the skills because of design challenges, and improved the participant’s trust with the technology, issues that had been previously identified as barriers to technology use among older adults [11,13].

Mrs B also found the *My Mantra* app to be helpful in changing her negative thoughts. When the coach first asked how she liked the app, she replied that, “It’s different.” The coach acknowledged that the skills may be different from what Mrs B was familiar with practicing in the other apps and encouraged her to give it a chance. Four days later, Mrs B reported that *My Mantra* was “growing on” her and was helping her to become more positive; however, she noted that “the camera part doesn’t work.” Through a back and forth SMS text message exchange, the coach determined that there was a *bug* in the app, and impressively, Mrs B had found a workaround and continued to use the app often. Despite the barrier of usability challenges [12,13], Mrs B continued to be successful in using and benefiting from the app.

Through interactions with the coach, Mrs B made clear the many ways that she was making progress. She was able to easily identify the benefits of each app and expressed that the different skills helped her see the fallacies behind her negative thoughts, recognize patterns, redirect her focus, and change negative thoughts into positive ones. During her second week, when the coach asked what she thought of the assigned app for that week (*Day to Day*), she said that it helped her to notice her negative thoughts and differentiate between the reality of the thoughts and the negative thought patterns that contributed to her symptoms of anxiety and depression:

The day to day makes me think about the negative thoughts and what do they really mean or is my mind trying to make me feel bad about my life and the past.

During week 7, she also shared with the coach that she was able to successfully follow through taking action toward suggestions made by *Day to Day*, such as talking to a friend.

At screening, Mrs B had elevated anxiety, with a GAD-7 score of 8, indicating mild anxiety. When she began the intervention, her GAD-7 score was 4. After completing the intervention, her GAD-7 anxiety score was 0. Her depression scores also dropped from 9 at screening and 8 at onboarding to 1 following treatment.

During her final week, when reflecting on her progress with treatment, she said:

The negative thoughts do not occur frequently like when I started the program. There was one issue that has been resolved and I can control the thought and not feel any resentment about the situation. It would trouble me daily and that's much better.

When asked about the most helpful skill that she learned in the intervention, she referred to the *Thought Challenger*:

The steps of changing negative thoughts to positive ones. I was plagued by negative thoughts for almost a year daily.

Her ability to implement the skills to change maladaptive cognitions is an indication of her successes using the apps and coaching to reach her goals and improve her depression.

### Case Study 3

Participant C was a 62-year-old white woman who was married and retired. She reported having an associate degree (ie, a 2-year college degree). She began the program wishing that she could work again because she was lonely all day at home. She felt she had no purpose, and her self-worth was low. She also reported experiencing migraines and insomnia, and she described a dysfunctional and chaotic extended family dynamic. Her goals for treatment were to be a more effective and assertive communicator, get better sleep, have less anxiety, take medications less frequently when upset, and improve her self-worth.

She used the apps regularly, with 3 days being the longest gap without app use. Over the course of the program, she demonstrated high engagement with coaching and frequently initiated contact with the coach to report on her progress. In week 2, when the coach asked Mrs C how she was feeling, she described positive changes in her interactions with her daughter and husband, noting that after repeatedly reading the material in her 2 apps, *Day to Day* and *Daily Feats*, she had begun to think before she spoke. She said she was relearning how to act and react in situations.

The next day, she said she was proud to report that she had used one of the teaching tools to help an acquaintance who had expressed self-blame. Using the process described in the *Day to Day* app lessons, she helped him to see a negative pattern in his thoughts and better assess the reality of the situation. She said that this exchange made her feel empowered that she could help others. When the coach asked if she had used any of the skills to work on her feelings of loneliness, she reported several examples of initiating social activities and plans for future volunteer activities. This shows how IntelliCare helped to address older adults’ desire for human contact [11] through interactions with the coach to discuss plans and take actions as well as following the suggestions from the apps to be involved in socialization.

Mrs C did an effective job of communicating her app preferences to the coach, which enabled them to identify relevant apps on which to focus. When beginning the week of using the *My Mantra* app, she let the coach know that she did not like that app because she did not use the camera or photo functions on her phone. Consistent with the coaching protocol, which instructs coaches to help the participant stay engaged with something they find valuable rather than encourage use of an app they do not find appealing, the coach suggested Mrs C focus on a different app by asking which app was her favorite. Mrs C reported that she enjoyed using the *Thought Challenger* app and decided to focus on that app instead. She then described how *Thought Challenger* helped her remember that some of the negative things that her husband would say during arguments were not actually true. She was learning not to internalize these comments. In this example, Mrs C overcame the barrier of willingness to ask for help [13] and showed the ability to continue engaging in the intervention.

During the midpoint phone call, Mrs C mentioned the importance of the *Hub* app lessons, noting that reading them repeatedly helped her to better understand and implement her new skills. Mrs C found the lessons to be so beneficial that she wanted to put a segment of it up on her wall at home. She planned to create a plaque with the words “action before motivation” because she said she had learned that if she waits until she feels like doing something, she would never get anything done. Her strides toward goals were made evident by her reports of being assertive in conversations with her daughters, not engaging in arguments with her husband when he gets “grumbly,” and using nonessential medications less often. These examples of increased insight, behavioral improvement, and heightened enthusiasm were encouraging indications of progress.

At the end of week 6, Mrs C described a situation where she demonstrated an increased ability to disengage from negative feelings. She shared feeling hurt and left out when her sisters did not invite her to vacation with them. She reported that normally, this experience would have bothered her for a long time. However because of what she learned through *Worry Knot* and *Thought Challenger*, she said the situation was not going to get her down because she had several positive things in her life and she had been accomplishing goals each day. Instead, she said she was able to enjoy the weekend with her husband.

Finally, she shared positive progress in addressing her anxieties using *Worry Knot*. These skills made her feel stronger mentally and more at ease. She said that she no longer had to reach for a medication to calm down and instead used the skills from the app to think about more neutral situations such as brushing her teeth. This showed that she employed the app’s skill of relating her worry with a neutral thought. These examples demonstrate Mrs C’s trust in technology, a known barrier to technology use among older adults [13]. By continuously using the apps, she showed her trust that the apps would help her feel better.

As she began her final week of coaching, she shared that a year prior, she had been on the verge of suicide and said:

The intervention gives me tools to help myself [...] I am happy and sleeping again. I can refer back to the program and find self help […] I can talk to people and make sure they understand how I am feeling. I don't hide the hurt. This is the best thing that ever could have happened for me. I am a happy person setting goals everyday. I am a worthy person of friendship and love.

These statements show her substantial growth toward improved self-worth, better sleep, less medication use, and better communication.

Her assessment of her progress matched her symptom scores. At screening, Mrs C had elevated depression and anxiety, with PHQ-8 and GAD-7 scores of 17 and 13, respectively. These scores were in the moderately severe depression and moderate anxiety ranges. When she was onboarded to the intervention, her PHQ-9 and GAD-7 scores were 11 and 8, respectively. After completing the intervention, her PHQ-9 depression and GAD-7 anxiety scores were both 0, which showed that she progressed to full remission.

In the final days of her intervention, Mrs C further discussed her progress with her goals for treatment as well as her plans for the future in SMS text messages to the coach. Having already joined several clubs and found ways to be less lonely, she said she planned to continue using the apps and setting goals. She further noted:

I feel stronger mentally and realized you can't live from the past but grow on the knowledge of it. What has happened in life has happened and to keep going forward and not back. [...] I don't focus on the past and do the “what if's.”

Mrs C began the program with low opinions about herself and a dim outlook on her life in general. As the intervention progressed, the coach watched as Mrs C changed her perception and applied the skills to her daily life. As made evident through her SMS text message correspondence, Mrs C reached all of her goals and had concrete plans to maintain them after coaching ended.

## Discussion

### Principal Findings

Previous studies have shown that multiple barriers might lead older adults to avoid using technology to support mental health, including a desire for human contact, complexity, trust in the technology, and design challenges [11-13]. In this study, we presented case studies of 3 individuals over the age of 60 years who fared well with a digital mental health intervention in terms of engagement with the technology and coaching, and progress toward their goals. Despite potential barriers and experiencing some technical glitches, these participants showed proficient ability to use the apps, high levels of participation through frequent app use and coach interaction, and decreased symptom scores through the intervention.

The 3 cases also demonstrated how different users have different ways of learning, and therefore, engage with the apps in unique ways. These individual preferences for different learning and engagement methods were accommodated through the use of a platform that contained multiple apps. For example, Mrs C found that reading the psychoeducational content was most helpful for her, whereas Mr A connected better with the app that gave him short and simple lists of goals that he could complete daily and check off his list. At the end of the 8-week intervention, all 3 participants expressed great enthusiasm for the benefit of this program through feedback to their coach, and they each identified a number of ways they had seen improvements in themselves.

There may be several reasons why these 3 participants performed well with the intervention. The first may be the benefit of having a coach who encouraged participants to use the apps over 8 weeks. Human contact is a feature that older adults have previously indicated that they are seeking to address their mental health [11]. Future development of mobile mental health technology for older adults may benefit from the incorporation of human contact. Remote engagement using SMS text messaging and phone calls can better facilitate person-to-person contact, which cuts out the time and cost of travel, missed work because of appointments, and limited scheduling availability. SMS text messaging within this study was a noninvasive form of contact, which gave the participants the option to respond at their convenience, or not at all. The choice to engage was optional, which may have relieved the pressure to participate immediately and gave the participants the liberty to converse with the coach as their schedule allowed. The cost of managing coaching services is small but may be further mitigated by automating some aspects of the service (eg, SMS text messages that are routinely sent). The costs associated with managing this service may also be offset by avoiding more expensive mental health services.

Second, in this study, participants engaged with the apps over a much longer duration than in prior research on mental health technologies among older adults, which focused on first impression encounters of the technologies over a few hours [11]. As seen with the apps that had glitches, the 3 case study participants were able to take the time to work through the problems and continued using the apps regardless.

Issues with complexity, familiarity, willingness to ask for help, trust in the technology, privacy, and design challenges have also been found to be a hindrance to older adults’ use of technology to manage their health [12,13]. IntelliCare addresses design challenges by implementing simple-to-use apps that take very little time to complete. The participants were repeatedly prompted to ask for help through noninvasive SMS text messages from their coach. The coach was unable to see any information entered into the apps, which provided privacy and may have increased trust with the intervention. Participants were urged to use each app daily to increase familiarity, and the coach provided accountability and encouragement to continue use. Any trouble they encountered could be discussed with the coach.

### Limitations

This study is intended to demonstrate the processes of coaching in older adults using a digital mental health service. Given that there are only 3 cases, these findings may not be generalizable to all older adults. For example, these participants each had some amount of college education, which may have contributed to their success with the intervention. We did not collect data regarding their level of previous experience with mobile technology, which may vary considerably in this population.

### Conclusions

These 3 cases provide examples of older individuals who engaged with and benefitted from the IntelliCare service. Although results from the 3 cases may not generalize to others, they provide an important, informed perspective of the experiences that can contribute to our understanding of how older adults use and overcome barriers to mental health technologies. These 3 cases offer detailed usability feedback for future intervention and technology design. Although this case study cannot determine whether all older adults can overcome these barriers, perhaps it may encourage and inform continued development of mobile mental health services for older adults. Evaluating intervention use metrics and clinical outcomes among the full subset of older adults in this study represents an important next step.

## Abbreviations

GAD-7: 7-item Generalized Anxiety Disorder scale
PHQ-8: Patient Health Questionnaire-8
UAMS: University of Arkansas for Medical Sciences
